# Supplementary material for: A single mutation in Crimean-Congo hemorrhagic fever virus discovered in ticks impairs infectivity in human cells
Source: eLife. 2020 Oct 21;9:e50999. doi: 10.7554/eLife.50999 (PMC7652417; doi:10.7554/eLife.50999)
Supplement: Supplementary file 1. — In parentheses is information about the country and year of strain isolation, the clade into which the strain groups, and the host from which it was isolated. Numbers represent length of the complete nucleotide and amino acid sequences, or lengths of particular domains/proteins, positions of the domains/proteins in the complete amino acid sequence, and pairwise identity of the sequences compared to MTBG2012-T1303. [file elife-50999-supp1.docx]

Supplementary File 1. Differences between strain Malko Tarnovo from tick T1303 (MT-BG2012-T1303) and pathogen strains of other lineages. In parentheses is information about the country and year of strain isolation, the clade into which the strain groups, and the host from which it was isolated. Numbers represent length of the complete nucleotide and amino acid sequences, or lengths of particular domains/proteins, positions of the domains/proteins in the complete amino acid sequence, and pairwise identity of the sequences compared to MT-BG2012-T1303.

| Seg.* |  |  | Strain MT-BG2012-T1303 (Bulgaria 2012; Europe 2; *Rhipicephalus bursa*) | Strain AP92 (Greece 1975; Europe 2; *R. bursa*) | Strain Pentalofos (Greece 2015; Europe 2; *R. bursa)* | Strain Kosovo Hoti (Kosovo 2001; Europe 1; *Homo sapiens*) | Strain ArD8194 (Senegal 1969; Africa 1; *Hyalomma truncatum*) | Strain Nakiwogo (Uganda 1958; Africa 2; *H. sapiens*) | Strain IbAr10200 (Nigeria 1996; Africa 3; *Hyalomma excavatum*) | Strain Matin (Pakistan 1976; Asia 1; *Homo sapiens*) | Strain NIV 112143 (India 2011; Asia 2; *Homo sapien*s) |
| --- | --- | --- | --- | --- | --- | --- | --- | --- | --- | --- | --- |
| S | ORF | nt | 1449 | 1449/ 91.9% | 1449/ 96.5% | 1449/ 83.1% | 1449/  82.5% | 1449/ 82.1% | 1449/  81.8% | 1449/ 82.7% | 1449/ 83.4% |
|  |  | aa | 482 | 482/ 99.4% | 482/ 99.2% | 482/ 93.0% | 482/ 92.5% | 482/ 92.5% | 482/ 91.9% | 482/ 92.5% | 482/ 92.3% |
|  | Positive sense ORF (NS_s_) | nt | 282/ 693–412 | 159/ 536–378/ 93.1% | 282/ 693–412/ 98.2% | 477/ 851–375/ 81.9% | 477/ 851–375/ 81.9% | 471/ 906–430/ 81.9% | 453/ 882–430/ 81.9% | 453/ 827–375/ 81.9% | 405/ 504–908/ 78.9% |
|  |  | aa | 93 | 53/ 94.3% | 93/ 100% | 159/ 84.0% | 159/ 87% | 159/ 77.9% | 150/ 78.7% | 151/ 78.7% | 135/ 78.9% |
| M | ORF | nt | 5088 | 5088/ 86.9% | 5088/ 96.3% | 5067/ 72.9% | 5103/ 72.7% | 5100/ 72.6% | 5055/ 72.5% | 5070/ 72.1% | 5055/ 73.1% |
|  |  | aa | 1695 | 1695/ 89.9% | 1695/ 97.3% | 1688/ 74.3% | 1700/ 75.1% | 1699/ 73.5% | 1684/ 74.5% | 1689/ 75.2% | 1684/ 74.4% |
|  | ORF Mucin-variable region | aa | 228/ 27–254 | 228/ 27–254/ 60.1% | 228/ 27–254/ 92.1% | 247/ 30–247/ 29.3% | 234/ 26–259/ 27.9% | 241/ 18–258/ 26.3% | 222/ 21–243/ 26.7% | 222/ 27–248/ 26.8% | 219/ 25–243/ 26.3% |
|  | Cleavage site | aa | RKLL 157–160; RSKR | RKLL 157–160; RSKR | RKLL 157–160; RSKR | RSKL | RSKR | RSKR | RSKR | RSKR | RSKR |
|  | ORF GP38 | aa | 268/ 259–526 | 268/ 259–526/ 92.9% | 268/ 259–526/ 97% | 268/ 252–519/ 70.5% | 168/ 264–531/ 75.4% | 262/ 263–531/ 74.3% | 268/ 248–515/ 71.3% | 268/ 253–520/ 75.0% | 268/ 248–515/ 73.9% |
|  | Cleavage site | aa | RRLL | RRLL | RRLL | RKLL | RRLL | RRLL | RRLL | RRLL | RRLL |
|  | ORF Gn | aa | 284/ 531–814 | 284/ 531–814/ 97.5% | 284/ 531–814/ 99.6% | 284/ 524–807/ 86.3% | 284/ 536–819/ 84.5% | 284/ 535–818/ 84.5% | 284/ 520–803/ 88.0% | 284/ 525–808/ 86.3% | 284/ 520–803/ 87.3% |
|  | Cleavage site | aa | RKLL | RKLL | RKLL | RKLL | RKLL | RRLL | RKLL | RKLL | RKLL |
|  | ORF NS_M_ | aa | 229/ 819–1047 | 229/ 819–1047/ 89.1% | 229/ 819–1047/ 97.8% | 229/ 812–1040/ 72.9% | 229/ 824–1052/ 76.0% | 229/ 823–1051/ 74.2% | 229/ 808–1036/ 72.1% | 229/ 813–1041/ 72.1% | 229/ 808–1036/ 70.3% |
|  | Cleavage site | aa | RKPL | RKPL | RKPL | RKPL | RKPL | RKPL | RKPL | RKPL | RRPL |
|  | ORF Gc | aa | 644/ 1052–1695 | 644/ 1052–1695/ 96.7% | 644/ 1052–1695/ 98.1% | 644/ 1045–1688/ 82.2% | 644/ 1057–1700/ 89.0% | 644/ 1067–1699/ 87.0% | 644/ 1041–1684/ 88.7% | 645/ 1045–1685/ 89.4% | 644/ 1041–1684/ 89.3% |
| L | ORF | nt | 11838 | 11838/ 96.2% | 11838/ 96.2% | 11838/ 78.4% | 11838/ 78.4% | 11838/ 78.2% | 11838/ 78.3% | 11838/ 78.7% | 11838/ 78.8% |
|  |  | aa | 3945 | 3945/ 98.8% | 3945/ 98.7% | 3945/ 90.8% | 3945/ 90.7% | 3945/ 90.5% | 3945/ 90.7% | 3945/ 91.0% | 3945/ 91.4% |
|  | ORF OTU | aa | 118/ 35–152 | 118/ 35–152/ 99.2% | 118/ 35–152/ 99.2% | 118/ 35–152/ 89.0% | 118/ 35–152/ 88.1% | 118/ 35–152/ 87.3% | 118/ 35–152/ 87.3% | 118/ 35–152/ 89.8% | 118/ 35–152/ 89.8% |
|  | ORF RdRp catalytic domain | aa | 672/ 2043–2714 | 672/ 2043–2714/ 99.6% | 672/ 2043–2714/ 99.1% | 672/ 2043–2714/ 94.5% | 672/ 2043–2714/ 94.9% | 672/ 2043–2714/ 94.6% | 672/ 2043– 2714/ 94.8% | 672/ 2043–2714/ 95.1% | 672/ 2043–2714/ 95.8% |

* Segment
