# Supplementary material for: A single mutation in Crimean-Congo hemorrhagic fever virus discovered in ticks impairs infectivity in human cells
Source: eLife. 2020 Oct 21;9:e50999. doi: 10.7554/eLife.50999 (PMC7652417; doi:10.7554/eLife.50999)
Supplement: Supplementary file 2. — Positions of substitutions are indicated by subscripted numbers. [file elife-50999-supp2.docx]

Supplementary File 2. Comparison of amino acid substitutions between protein sequences of MT-BG2012-T1303 (accession numbers MK299341, MK299342, MK299343) and AP92 (accession numbers DQ211638, DQ211625, DQ211612), as well as between MT-BG2012-T1303 and strains comprissing Europe 1 lineage (Kosovo Hoti, accession numbers DQ133507, EU037902, EU044832; Turkey200310849, accession numbers DQ211649, DQ211636, DQ211623; Turkey-Kelkit06, accession numbers GQ337053, GQ337054, GQ337055; Drosdov, accession numbers DQ211643, DQ211630, DQ211617; Kashmanov, accession numbers DQ211644, DQ211631, DQ211618). Positions of substitutions are indicated by subscripted numbers.

| S segment | | | M segment | | | L segment | | |
| --- | --- | --- | --- | --- | --- | --- | --- | --- |
| MT-BG2012-T1303 Europe 1 | MT-BG2012-T1303- AP92 | MT-BG2012-T1303-  Pentafolos | MT-BG2012-T1303- Europe 1 | MT-BG2012-T1303- AP92 | MT-BG2012-T1303-  Pentafolos | MT-BG2012-T1303-Europe 1 | MT-BG2012-T1303- AP92 | MT-BG2012-T1303- Pentafolos |
| D_12_-E | T_65_-S | T_65_-S | S_2_-P | S_2_-P | M_6_-T | E_2_-D | V_114_-I | V_114_-I |
| L_13_-M | F_73_-Y | S_125_-G | D_3_-I | M_6_-I | H_16_-L | S_6_-N | T_165_-A | T_165_-A |
| V_27_-M | A_271_-T | N_275_-S | H_4_-N | F_7_-L | T_31_-S | L_13_-I | Y_495_-F | R_218_-K |
| R_45_-K |  | N_475_-S | F_5_-I | I_11_-M | G_37_-S | S_24_-N | M_505_-T | E_264_-D |
| Y_48_-F |  |  | F_7_-H | C_12_-Y | V_38_-A | D_55_-A | G_593_-S | S_477_-T |
| T_65_-S |  |  | I_11_-V | L_15_-F | K_54_-R | I_64_-L | D_787_-E | I_480_-T |
| T_100_-S |  |  | T_14_-F | K_17_-Q | Q_55_-L | K_66_-E | A_931_-T | Y_495_-F |
| S_101_-G |  |  | H_16_-Y | S_18_-H | P_56_-A | T_67_-L | V_1008_-I | S_647_-N |
| S_109_-N |  |  | K_17_-L | G_21_-D | I_59_-T | Q_70_-R | V_1023_-I | K_699_-R |
| I_117_-V |  |  | S_18_-Q | T_26_-V | Q_64_-H | L_74_-Q | D_1041_-E | G_768_-E |
| S_124_-A |  |  | P_19_-L | S_30_-N | A_92_-V | D_75_-E | S_1064_-N | P_870_-S |
| S_125_-N |  |  | Q_20_-L | T_31_-P | G_100_-S | Q_91_-K | S_1089_-N | A_931_-T |
| I_141_-V |  |  | V_21_-L | G_33_-N | Y_113_-H | S_94_-L | D_1163_-E | M_953_-K |
| T_146_-A |  |  | S_23_-- | N_34_-S | S_116_-G | V_114_-I | I_1180_-V | V_1023_-I |
| L_163_-V |  |  | A_24_-G | V_38_-A | L_172_-S | T_122_-A | D_1194_-E | D_1041_-E |
| V_164_-M |  |  | N_25_-G | G_39_-S | T_174_-A | N_124_-S | R_1222_-K | S_1064_-N |
| P_165_-S |  |  | T_26_-A | S_44_-P | Q_188_-P | E_135_-D | F_1225_-L | S_1068_-N |
| I_179_-L |  |  | L_28_-G/R | V_48_-A | H_210_-S | V_157_-I | T_1279_-A | D_1079_-N |
| I_198_-V |  |  | V_29_-Q | N_51_-D | S_211_-N | L_161_-F | A_1288_-T | S_1089_-N |
| I_205_-V |  |  | T_31_-N | S_52_-G | A_229_-D | G_167_-E | T_1449_-A | D_1163_-E |
| E_248_-D |  |  | S_32_-A/T | P_56_-S | Y_325_-H | T_168_-A | R_1478_-K | F_1225_-L |
| K_258_-N |  |  | G_33_-T | I_59_-T | N_336_-S | P_189_-L | I_1502_-V | V_1256_-I |
| D_259_-G |  |  | N_34_-E | P_61_-T | A_346_-V | A_200_-T | A_1589_-V | K_1272_-R |
| N_275_-S |  |  | T_35_-H | Q_64_-R | N_347_-S | D_201_-S | K_1680_-R | T_1279_-A |
| V_277_-I |  |  | T_36_-N | L_65_-P | S_360_-R | T_202_-A/V | I_1695_-V | A_1288_-T |
| S_279_-N |  |  | V_38_-T | G_66_-S | S_381_-T | R_206_-Y | I_1784_-V | I_1502_-V |
| N_419_-S |  |  | G_39_-N | T_71_-I | A_388_-V | T_209_-S | I_1865_-V | I_1865_-V |
| S_422_-T |  |  | A_41_-- | I_72_-T | I_454_-L | R_219_-K | I_1909_-V | I_1909_-V |
| R_428_-K |  |  | F_42_-- | E_73_-T | G_652_-R | V_223_-I/L | T_1964_-A | T_1964_-A |
| D_442_-N |  |  | N_43_-- | T_77_-M | Y_868_-F | T_262_-S | N_1978_-S | Q_1981_-L |
| K_444_-Q |  |  | S_44_-T | A_79_-V | G_878_-S | S_263_-A | D_1989_-N | I_2100_-V |
|  |  |  | V_45_-T | S_86_-T | T_955_-A | E_264_-D | G_2015_-E | R_2127_-K |
|  |  |  | H_46_-A | S_89_-P | I_1046_-M | M_267_-T | K_2128_-R | G_2130_-S |
|  |  |  | N_47_-P | A_92_-T | L_1047_-S | H_306_-Y | I_2438_-T | I_2141_-V |
|  |  |  | V_48_-G/S | S_93_-P | G_1116_-R | V_352_-I | R_2531_-K | I_2355_-V |
|  |  |  | T_50_-S | T_97_-E | M_1129_-I | F_385_-Y | S_2715_-N | R_2531_-K |
|  |  |  | N_51_-Q | V_98_-T | I_1331_-V | S_390_-N | I_2721_-V | A_2733_-T |
|  |  |  | Q_53_-P | G_100_-T | I_1351_-V | S_392_-G | A_2733_-T | K_3047_-R |
|  |  |  | Q_55_-P | S_103_-N | F_1370_-Y | T_405_-A | R_3161_-K | R_3150_-K |
|  |  |  | T_57_-A/V/M | W_104_-Q | I_1435_-V | I_406_-T | I_3376_-V | R_3161_-K |
|  |  |  | I_59_-T | R_105_-K | S_1494_-N | N_453_-S | R_3537_-K | I_3376_-V |
|  |  |  | M_62_-S/P | P_106_-S | R_1544_-E | Y_495_-F | T_3578_-S | M_3433_-L |
|  |  |  | A_63_-H | T_107_-A | L_1611_-F | D_498_-S | I_3705_-M | R_3537_-K |
|  |  |  | Q_64_-A | V_110_-M | K_1650_-R | S_499_-D | N_3777_-S | D_3560_-G |
|  |  |  | G_66_-E | N_111_-G | R_1663_-K | H_502_-R | K_3798_-R | T_3578_-S |
|  |  |  | T_67_-P/S | A_112_-T | F_1674_-L | C_504_-S | S_3816_-T | I3_581_-V |
|  |  |  | V_68_-S | Y_113_-L |  | M_505_-T | F_3883_-L | V_3703_-I |
|  |  |  | Q_70_-I | I_119_-D |  | S_507_-N | A_3898_-T | I_3705_-M |
|  |  |  | T_71_-K | T_121_-A |  | V_508_-I | S_3904_-F | S_3839_-G |
|  |  |  | I_72_-L/P | P_122_-L |  | T_509_-A |  | N_3854_-C |
|  |  |  | E_73_-T | T_123_-M |  | H_514_-F |  | A_3898_-T |
|  |  |  | A_74_-T | I_124_-T |  | T_556_-S |  | S_3904_-F |
|  |  |  | Q_75_-P | S_126_-T |  | S_561_-T |  |  |
|  |  |  | T_77_-S | D_127_-G |  | R_599_-K |  |  |
|  |  |  | D_80_-E | K_128_-R |  | S_674_-N |  |  |
|  |  |  | S_85_-T | G_129_-S |  | E_676_-D |  |  |
|  |  |  | S_86_-- | T_130_-I |  | V_715_-I |  |  |
|  |  |  | L_88_-P/S | P_131_-L |  | A_719_-V |  |  |
|  |  |  | S_89_-P/T | L_133_-S |  | C_761_-H |  |  |
|  |  |  | A_90_-T/P | S_138_-G |  | D_762_-G |  |  |
|  |  |  | V_91_-N | L_139_-S |  | A_763_-G |  |  |
|  |  |  | A_92_-T | S_140_-F |  | P_765_-L |  |  |
|  |  |  | S_93_-T | S_141_-D |  | G_767_-A/T |  |  |
|  |  |  | T_94_-Q | K_142_-R |  | G_768_-K |  |  |
|  |  |  | P_95_-D/G | N_144_-S |  | K_769_-E |  |  |
|  |  |  | A_96_-S/P/L | R_145_-K |  | E_770_-K |  |  |
|  |  |  | T_97_-P/S | S_147_-Q |  | G_771_-E/D |  |  |
|  |  |  | V_98_-P/S | H_153_-N |  | T_772_-G |  |  |
|  |  |  | T_99_-L/P | A_163_-V |  | E_773_-D |  |  |
|  |  |  | G_100_-E | T_167_-A |  | Q_774_-L |  |  |
|  |  |  | H_101_-T/A | V_168_-A |  | G_776_-E |  |  |
|  |  |  | A_102_-T | S_169_-P |  | P_778_-G |  |  |
|  |  |  | W_104_-E | L_172_-S |  | T_779_-N/S |  |  |
|  |  |  | S_108_-T | S_177_-T |  | S_780_-Q |  |  |
|  |  |  | V_110_-A/S | T_178_-A |  | G_782_-S |  |  |
|  |  |  | N_111_-T/I | E_179_-D |  | T_783_-A |  |  |
|  |  |  | A_112_-S | G_180_-Q |  | D_784_-V |  |  |
|  |  |  | Y_113_-- | T_183_-A |  | P_786_-H |  |  |
|  |  |  | T_115_-S/P | A_185_-T |  | D_787_-E |  |  |
|  |  |  | L_117_-T | A_189_-V |  | T_789_-P |  |  |
|  |  |  | E_118_-D | P_194_-S |  | N_790_-S |  |  |
|  |  |  | I_119_-N/S | T_195_-P |  | V_791_-I |  |  |
|  |  |  | T_121_-N | A_196_-S |  | L_792_-S |  |  |
|  |  |  | I_124_-T | A_197_-T |  | S_793_-A/V |  |  |
|  |  |  | L_125_-Q/H | T_201_-V |  | R_798_-G |  |  |
|  |  |  | S_126_-M/V | P_204_-S |  | F_810_-L |  |  |
|  |  |  | D_127_-T | V_205_-A |  | K_818_-T |  |  |
|  |  |  | K_128_-D | D_206_-G |  | K_876_-T |  |  |
|  |  |  | G_129_-N | P_209_-S |  | K_879_-R |  |  |
|  |  |  | Q_134_-T | H_210_-S |  | I_882_-V |  |  |
|  |  |  | S_135_-V/I | S_211_-N |  | A_931_-T |  |  |
|  |  |  | Q_136_-S | P_212_-S |  | S_935_-L |  |  |
|  |  |  | K_142_-S | T_216_-S |  | G_943_-E |  |  |
|  |  |  | T_143_-P | P_222_-L |  | S_947_-N |  |  |
|  |  |  | N_144_-S | P_225_-S |  | M_953_-T |  |  |
|  |  |  | R_145_-T/M | S_226_-G |  | N_956_-A |  |  |
|  |  |  | R_150_-Q | P_227_-S |  | N_957_-S |  |  |
|  |  |  | S_151_-G | S_239_-N |  | L_979_-S |  |  |
|  |  |  | T_152_-I | A_244_-V |  | M_988_-L |  |  |
|  |  |  | Q_154_-H | I_245_-T |  | Q_997_-R |  |  |
|  |  |  | L_156_-V/A | T_248_-A |  | Y_1000_-F |  |  |
|  |  |  | K_158_-S/N | R_278_-K |  | V_1009_-T |  |  |
|  |  |  | S_169_-T | T_305_-A |  | T_1020_-S |  |  |
|  |  |  | A_163_-S | S_309_-G |  | V_1023_-I |  |  |
|  |  |  | Q_170_-T | K_333_-R |  | A_1026_-S |  |  |
|  |  |  | T_171_-P | L_334_-S |  | S_1029_-T |  |  |
|  |  |  | L_172_-T | N_336_-S |  | N_1033_-T |  |  |
|  |  |  | A_175_-S | L_343_-S |  | A_1034_-N |  |  |
|  |  |  | V_176_-P | N_347_-S |  | I_1039_-V |  |  |
|  |  |  | S_177_-G | T_348_-P |  | E_1040_-D |  |  |
|  |  |  | T_178_-E | S_360_-K |  | D_1041_-E |  |  |
|  |  |  | E_179_-M | V_364_-I |  | T_1043_-A |  |  |
|  |  |  | G_180_-S | A_388_-I |  | M_1056_-L |  |  |
|  |  |  | T_181_-S | A_393_-I |  | T_1059_-V |  |  |
|  |  |  | A_185_-S | I_395_-V |  | G_1063_-S |  |  |
|  |  |  | S_186_-Q | R_420_-K |  | S_1064_-N |  |  |
|  |  |  | P_187_-H | I_454_-L |  | S_1068_-N |  |  |
|  |  |  | Q_188_-S | I_465_-V |  | N_1073_-S |  |  |
|  |  |  | T_190_-M | R_501_-K |  | D_1079_-C |  |  |
|  |  |  | T_191_-S | I_503_-V |  | C_1084_-K |  |  |
|  |  |  | D_192_-R | A_533_-G |  | V_1085_-I |  |  |
|  |  |  | A_196_-- | S_535_-D |  | R_1094_-K |  |  |
|  |  |  | A_197_-P/L | R_546_-K |  | I_1124_-V |  |  |
|  |  |  | P_198_-H/Y | S_603_-N |  | K_1156_-R |  |  |
|  |  |  | Q_200_-A | Y_609_-A |  | A_1158_-S |  |  |
|  |  |  | T_202_-Q/R | G_652_-K |  | L_1161_-H |  |  |
|  |  |  | S_203_-V | K_794_-R |  | K_1162_-N |  |  |
|  |  |  | P_204_-S | M_826_-V |  | S_1165_-T/N |  |  |
|  |  |  | V_205_-T | I_836_-V |  | R_1167_-K |  |  |
|  |  |  | D_206_-E | I_843_-V |  | K_1169_-R |  |  |
|  |  |  | S_207_-N/I | G_854_-S |  | E_1172_-D |  |  |
|  |  |  | S_208_-A/T | A_858_-T |  | N_1173_-D |  |  |
|  |  |  | P_209_-N | F_869_-S |  | R_1179_-K |  |  |
|  |  |  | N_214_-R | F_874_-V |  | I_1182_-T |  |  |
|  |  |  | H_215_-Q | A_875_-I |  | K_1184_-R |  |  |
|  |  |  | T_216_-S | G_878_-S |  | A_1190_-S |  |  |
|  |  |  | K_217_-E | K_888_-R |  | K_1200_-R |  |  |
|  |  |  | T_218_-S | I_904_-V |  | I_1210_-L |  |  |
|  |  |  | T_219_-S | S_926_-G |  | R_1215_-K |  |  |
|  |  |  | P_220_-AV | E_941_-D |  | F_1225_-L |  |  |
|  |  |  | S_224_-T | R_952_-K |  | Y_1273_-C |  |  |
|  |  |  | E_228_-V/M/I | T_955_-A |  | A_1276_-V |  |  |
|  |  |  | A_229_-T | A_972_-I |  | H_1277_-Q |  |  |
|  |  |  | Q_230_-S | G_976_-S |  | T_1278_-S |  |  |
|  |  |  | Q_231_-P | R_978_-K |  | T_1279_-S/F |  |  |
|  |  |  | T_232_-A | L_981_-F |  | R_1285_-K |  |  |
|  |  |  | P_234_-S | I_985_-V |  | A_1288_-V |  |  |
|  |  |  | T_235_-I | M_992_-V |  | H_1308_-Y |  |  |
|  |  |  | T_236_-L | V_999_-A |  | M_1309_-I |  |  |
|  |  |  | M_237_-L/V | T_1002_-A |  | S_1310_-G |  |  |
|  |  |  | P_238_-M | T_1005_-A |  | V_1317_-I |  |  |
|  |  |  | T_240_-A | Y_1045_-F |  | V_1331_-T |  |  |
|  |  |  | A_242_-P | T_1092_-A |  | I_1358_-V |  |  |
|  |  |  | L_243_-T/I | G_1116_-R |  | N_1389_-G |  |  |
|  |  |  | P_246_-Q | M_1129_-I |  | K_1395_-Q |  |  |
|  |  |  | T_247_-N/D | V_1290_-I |  | V_1416_-I |  |  |
|  |  |  | T_248_-I | E_1298_-D |  | S_1436_-N |  |  |
|  |  |  | I_250_-P | I_1351_-V |  | D_1437_-E |  |  |
|  |  |  | S_260_-L | I_1405_-T |  | M_1439_-T |  |  |
|  |  |  | P_269_-S | I_1453_-V |  | S_1450_-L |  |  |
|  |  |  | S_277_-G | G_1502_-S |  | L_1455_-F |  |  |
|  |  |  | R_278_-K | N_1543_-K |  | A_1460_-F |  |  |
|  |  |  | S_291_-T | R_1544_-N |  | T_1461_-A |  |  |
|  |  |  | T_299_-K | D_1546_-E |  | A_1468_-E |  |  |
|  |  |  | T_301_-N | K_1566_-T |  | S_1474_-T |  |  |
|  |  |  | K_303_-G | T_1579_-S |  | A_1475_-S |  |  |
|  |  |  | Q_304_-S | K_1580_-R |  | R_1478_-K |  |  |
|  |  |  | T_305_-N | N_1597_-S |  | V_1495_-I |  |  |
|  |  |  | S_309_-D | I_1607_-F |  | F_1496_-V |  |  |
|  |  |  | Y_310_-F | L_1611_-F |  | I_1502_-V |  |  |
|  |  |  | E_313_-K | L_1622_-F |  | M_1507_-I |  |  |
|  |  |  | K_316_-E | S_1666_-N |  | G_1540_-N |  |  |
|  |  |  | L_320_-I/M | F_1674_-L |  | T_1565_-S |  |  |
|  |  |  | L_331_-Q |  |  | V_1566_-I |  |  |
|  |  |  | L_334_-T |  |  | E_1575_-D |  |  |
|  |  |  | N_336_-S |  |  | V_1577_-I |  |  |
|  |  |  | Q_337_-T |  |  | L_1582_-Q |  |  |
|  |  |  | G_339_-S |  |  | K_1583_-R |  |  |
|  |  |  | T_340_-S/P |  |  | D_1588_-E |  |  |
|  |  |  | G_342_-E |  |  | A_1589_-V |  |  |
|  |  |  | L_343_-P |  |  | V_1601_-I |  |  |
|  |  |  | P_344_-S |  |  | R_1655_-K |  |  |
|  |  |  | L_345_-H |  |  | E_1658_-A |  |  |
|  |  |  | N_347_-R/G |  |  | M_1682_-L |  |  |
|  |  |  | T_348_-L/P |  |  | L_1684_-S |  |  |
|  |  |  | F_357_-Y |  |  | S_1685_-N |  |  |
|  |  |  | Y_358_-F |  |  | D_1686_-S |  |  |
|  |  |  | S_360_-K |  |  | A_1688_-T |  |  |
|  |  |  | V_364_-S |  |  | N_1693_-Q |  |  |
|  |  |  | M_365_-I |  |  | V_1749_-I |  |  |
|  |  |  | K_377_-R |  |  | S_1758_-N |  |  |
|  |  |  | A_378_-S/L |  |  | D_1760_-E |  |  |
|  |  |  | N_380_-T/A |  |  | V_1762_-I |  |  |
|  |  |  | A_388_-V |  |  | V_1787_-I |  |  |
|  |  |  | S_392_-P |  |  | Y_1795_-F |  |  |
|  |  |  | K_394_-R |  |  | K_1910_-R |  |  |
|  |  |  | V_396_-A |  |  | E_1914_-D |  |  |
|  |  |  | N_397_-E |  |  | V_1932_-I |  |  |
|  |  |  | Y_403_-T |  |  | I_1949_-V |  |  |
|  |  |  | S_404_-T |  |  | D_1957_-E |  |  |
|  |  |  | N_405_-S |  |  | I_1960_-L |  |  |
|  |  |  | V_409_-I |  |  | E_1963_-S |  |  |
|  |  |  | K_415_-R |  |  | T_1964_-E |  |  |
|  |  |  | V_416_-A |  |  | Q_1967_-N |  |  |
|  |  |  | T_418_-V |  |  | E_1982_-D |  |  |
|  |  |  | D_419_-F |  |  | N_1987_-K |  |  |
|  |  |  | R_420_-K |  |  | A_1994_-S |  |  |
|  |  |  | D_421_-E |  |  | T_1996_-I |  |  |
|  |  |  | S_423_-R |  |  | S_1998_-C |  |  |
|  |  |  | L_425_-V/I |  |  | T_2001_-A |  |  |
|  |  |  | V_427_-I |  |  | N_2007_-S |  |  |
|  |  |  | V_434_-I |  |  | Q_2018_-H |  |  |
|  |  |  | I_438_-L |  |  | G_2025_-D |  |  |
|  |  |  | L_444_-V |  |  | V_2067_-I |  |  |
|  |  |  | K_446_-N |  |  | L_2105_-S |  |  |
|  |  |  | I_454_-L |  |  | P_2112_-T |  |  |
|  |  |  | M_459_-P |  |  | S_2124_-C |  |  |
|  |  |  | I_460_-V |  |  | G_2130_-D/Y |  |  |
|  |  |  | Q_464_-R/H |  |  | E_2131_-N/K |  |  |
|  |  |  | T_466_-Y |  |  | V_2134_-T |  |  |
|  |  |  | M_492_-L |  |  | Q_2135_-N |  |  |
|  |  |  | T_493_-V |  |  | N_2136_-S/G |  |  |
|  |  |  | I_503_-V |  |  | G_2138_-S |  |  |
|  |  |  | T_509_-V |  |  | I_2141_-L |  |  |
|  |  |  | R_514_-K |  |  | G_2197_-S |  |  |
|  |  |  | R_518_-K |  |  | D_2383_-N |  |  |
|  |  |  | V_519_-I |  |  | V_2392_-T |  |  |
|  |  |  | Q_521_-K |  |  | K_2418_-R |  |  |
|  |  |  | M_522_-A |  |  | H_2428_-Y |  |  |
|  |  |  | S_523_-T |  |  | I_2438_-A |  |  |
|  |  |  | T_524_-S |  |  | T_2495_-A |  |  |
|  |  |  | R_528_-K |  |  | Y_2497_-F |  |  |
|  |  |  | A_533_-E |  |  | R_2501_-K |  |  |
|  |  |  | G_534_-P |  |  | M_2503_-L |  |  |
|  |  |  | S_535_-G |  |  | I_2508_-V |  |  |
|  |  |  | G_536_-D |  |  | R_2531_-K |  |  |
|  |  |  | G_537_-D |  |  | D_2532_-E |  |  |
|  |  |  | I_545_-L |  |  | A_2535_-S |  |  |
|  |  |  | V_552_-I |  |  | I_2586_-V |  |  |
|  |  |  | G_554_-D |  |  | I_2594_-M |  |  |
|  |  |  | D_556_-N |  |  | H_2614_-Q |  |  |
|  |  |  | E_562_-D |  |  | I_2628_-V |  |  |
|  |  |  | I_589_-V |  |  | S_2695_-H |  |  |
|  |  |  | Q_600_-T |  |  | G_2711_-S |  |  |
|  |  |  | S_603_-T |  |  | E_2713_-G/S |  |  |
|  |  |  | V_609_-T |  |  | R_2714_-C/G/S |  |  |
|  |  |  | Q_612_-K |  |  | A_2717_-T |  |  |
|  |  |  | Y_617_-F |  |  | S_2720_-T |  |  |
|  |  |  | S_622_-T |  |  | I_2721_-V |  |  |
|  |  |  | I_637_-V |  |  | D_2722_-E |  |  |
|  |  |  | V_641_-A |  |  | I_2727_-V |  |  |
|  |  |  | T_644_-S |  |  | Q_2735_-H |  |  |
|  |  |  | I_648_-V |  |  | A_2741_-S |  |  |
|  |  |  | G_652_-K |  |  | I_2757_-L |  |  |
|  |  |  | Q_670_-K |  |  | R_2767_-K |  |  |
|  |  |  | S_671_-D |  |  | I_2769_-T |  |  |
|  |  |  | V_674_-I |  |  | I_2774_-V |  |  |
|  |  |  | N_680_-H |  |  | V_2775_-I |  |  |
|  |  |  | L_719_-F/S |  |  | F_2818_-L |  |  |
|  |  |  | T_721_-A |  |  | N_2894_-S |  |  |
|  |  |  | I_725_-S |  |  | K_2989_-R |  |  |
|  |  |  | L_736_-I |  |  | S_3003_-G/S |  |  |
|  |  |  | S_752_-T |  |  | I_3109_-M |  |  |
|  |  |  | V_753_-A |  |  | R_3150_-K |  |  |
|  |  |  | E_782_-D |  |  | T_3157_-S |  |  |
|  |  |  | T_785_-A |  |  | S_3162_-N |  |  |
|  |  |  | N_789_-K/P/T |  |  | H_3169_-Q |  |  |
|  |  |  | K_794_-R |  |  | K_3173_-R |  |  |
|  |  |  | M_826_-V |  |  | G_3251_-S/C |  |  |
|  |  |  | M_834_-L |  |  | T_3257_-A |  |  |
|  |  |  | I_836_-V |  |  | P_3278_-S |  |  |
|  |  |  | I_839_-V |  |  | G_3288_-S |  |  |
|  |  |  | I_842_-T |  |  | T_3350_-S |  |  |
|  |  |  | I_843_-V |  |  | T_3353_-S |  |  |
|  |  |  | M_845_-L |  |  | V_3383_-I |  |  |
|  |  |  | I_853_-V |  |  | V_3405_-L/M |  |  |
|  |  |  | Q_855_-H |  |  | M_3433_-L |  |  |
|  |  |  | E_856_-G |  |  | N_3439_-S |  |  |
|  |  |  | R_857_-K |  |  | I_3470_-V |  |  |
|  |  |  | A_858_-T |  |  | R_3537_-K |  |  |
|  |  |  | V_859_-I |  |  | R_3577_-K |  |  |
|  |  |  | A_860_-E |  |  | I_3581_-V |  |  |
|  |  |  | L_861_-T |  |  | S_3585_-T |  |  |
|  |  |  | F_864_-T |  |  | E_3588_-I/V |  |  |
|  |  |  | D_867_-G |  |  | D_3595_-N |  |  |
|  |  |  | Y_868_-F |  |  | I_3607_-V |  |  |
|  |  |  | M_869_-T/A |  |  | K_3652_-R |  |  |
|  |  |  | G_870_-S |  |  | I_3678_-V/M |  |  |
|  |  |  | A_875_-M |  |  | S_3696_-A |  |  |
|  |  |  | M_876_-L |  |  | I_3705_-M |  |  |
|  |  |  | G_878_-S |  |  | Q_3709_-N |  |  |
|  |  |  | V_879_-I |  |  | T_3714_-V |  |  |
|  |  |  | L_881_-F |  |  | L_3734_-F |  |  |
|  |  |  | A_882_-I |  |  | V_3736_-A/T |  |  |
|  |  |  | L_885_-C |  |  | C_3738_-Y |  |  |
|  |  |  | I_891_-V |  |  | T_3741_-N |  |  |
|  |  |  | I_894_-V |  |  | R_3743_-C |  |  |
|  |  |  | S_896_-D/E |  |  | G_3747_-D |  |  |
|  |  |  | T_89_7-S |  |  | T_3754_-A |  |  |
|  |  |  | I_904_-V |  |  | G_3758_-S |  |  |
|  |  |  | R_906_-K |  |  | I_3760_-V |  |  |
|  |  |  | H_909_-S/T |  |  | S_3776_-N |  |  |
|  |  |  | L_928_-F |  |  | I_3782_-V |  |  |
|  |  |  | A_935_-H |  |  | I_3787_-N |  |  |
|  |  |  | T_938_-A |  |  | H_3793_-L |  |  |
|  |  |  | Q_940_-R |  |  | S_3801_-P |  |  |
|  |  |  | A_942_-I |  |  | I_3804_-V |  |  |
|  |  |  | F_945_-L |  |  | T_3806_-I |  |  |
|  |  |  | V_949_-I |  |  | E_3821_-D |  |  |
|  |  |  | R_952_-K |  |  | V_3823_-M |  |  |
|  |  |  | K_334_-R |  |  | T_3824_-S |  |  |
|  |  |  | A_972_-T |  |  | A_3830_-S |  |  |
|  |  |  | G_976_-S |  |  | F_3831_-L |  |  |
|  |  |  | N_977_-R |  |  | S_3839_-N |  |  |
|  |  |  | V_979_-A |  |  | L_3840_-F |  |  |
|  |  |  | N_984_-S |  |  | K_3857_-R |  |  |
|  |  |  | M_992_-I |  |  | K_3874_-R |  |  |
|  |  |  | V_996_-T |  |  | D_3875_-E |  |  |
|  |  |  | M_997_-L |  |  | F_3883_-L |  |  |
|  |  |  | V_998_-T |  |  | V_3884_-I |  |  |
|  |  |  | V_999_-I |  |  | R_3890_-K |  |  |
|  |  |  | I_1001_-V |  |  | R_3893_-K |  |  |
|  |  |  | T_1002_-V |  |  | I_3896_-T |  |  |
|  |  |  | S_1004_-T |  |  | A_3898_-T |  |  |
|  |  |  | T_1005_-S |  |  | P_3899_-Q |  |  |
|  |  |  | E_1022_-D |  |  | A_3900_-I |  |  |
|  |  |  | S_1044_-A |  |  | A_3901_-E |  |  |
|  |  |  | Y_1045_-L |  |  | P_3902_-H |  |  |
|  |  |  | A_1057_-V |  |  | E_3903_-K |  |  |
|  |  |  | V_1060_-M |  |  | S_3904_-D |  |  |
|  |  |  | A_1084_-V |  |  | C_3919_-S |  |  |
|  |  |  | Y_1088_-F |  |  | I_3921_-T |  |  |
|  |  |  | T_1092_-V |  |  | T_3922_-A |  |  |
|  |  |  | S_1094_-T |  |  | V_3923_-I |  |  |
|  |  |  | T_1100_-S |  |  | V_3924_-D |  |  |
|  |  |  | S_1114_-T |  |  | Q_3925_-L |  |  |
|  |  |  | G_1116_-R |  |  | S_3939_-G/S |  |  |
|  |  |  | M_1129_-I/V |  |  |  |  |  |
|  |  |  | N_1132_-D |  |  |  |  |  |
|  |  |  | R_1142_-K |  |  |  |  |  |
|  |  |  | T_1143_-L |  |  |  |  |  |
|  |  |  | I_1146_-V |  |  |  |  |  |
|  |  |  | V_1148_-I |  |  |  |  |  |
|  |  |  | N_1179_-D |  |  |  |  |  |
|  |  |  | Y_1193_-H |  |  |  |  |  |
|  |  |  | L_1221_-V |  |  |  |  |  |
|  |  |  | E_1224_-D |  |  |  |  |  |
|  |  |  | L_1232_-F |  |  |  |  |  |
|  |  |  | S_1267_-N |  |  |  |  |  |
|  |  |  | L_1289_-I |  |  |  |  |  |
|  |  |  | V_1290_-I |  |  |  |  |  |
|  |  |  | Y_1300_-F |  |  |  |  |  |
|  |  |  | M_1310_-L |  |  |  |  |  |
|  |  |  | V_1325_-I |  |  |  |  |  |
|  |  |  | I_1331_-V |  |  |  |  |  |
|  |  |  | V_1334_-I |  |  |  |  |  |
|  |  |  | L_1355_-F |  |  |  |  |  |
|  |  |  | F_1370_-Y |  |  |  |  |  |
|  |  |  | A_1393_-V |  |  |  |  |  |
|  |  |  | I_1405_-T |  |  |  |  |  |
|  |  |  | R_1429_-T |  |  |  |  |  |
|  |  |  | T_1454_-S |  |  |  |  |  |
|  |  |  | T_1462_-A |  |  |  |  |  |
|  |  |  | S_1464_-T |  |  |  |  |  |
|  |  |  | I_1498_-V |  |  |  |  |  |
|  |  |  | S_1503_-T |  |  |  |  |  |
|  |  |  | I_1510_-L |  |  |  |  |  |
|  |  |  | V_1514_-T |  |  |  |  |  |
|  |  |  | S_1523_-A |  |  |  |  |  |
|  |  |  | D_1527_-T |  |  |  |  |  |
|  |  |  | D_1537_-E |  |  |  |  |  |
|  |  |  | N_1540_-K |  |  |  |  |  |
|  |  |  | N_1543_-S |  |  |  |  |  |
|  |  |  | R_1544_-E |  |  |  |  |  |
|  |  |  | R_1549_-K |  |  |  |  |  |
|  |  |  | N_1552_-D |  |  |  |  |  |
|  |  |  | A_1553_-T |  |  |  |  |  |
|  |  |  | G_1578_-T |  |  |  |  |  |
|  |  |  | T_1579_-A |  |  |  |  |  |
|  |  |  | T_1581_-A |  |  |  |  |  |
|  |  |  | D_1586_-E |  |  |  |  |  |
|  |  |  | F_1588_-V |  |  |  |  |  |
|  |  |  | S_1600_-G |  |  |  |  |  |
|  |  |  | G_1601_-S |  |  |  |  |  |
|  |  |  | I_1602_-V |  |  |  |  |  |
|  |  |  | V_1606_-E/L |  |  |  |  |  |
|  |  |  | I_1607_-F |  |  |  |  |  |
|  |  |  | L_1608_-I |  |  |  |  |  |
|  |  |  | A_1618_-V |  |  |  |  |  |
|  |  |  | L_1621_-V |  |  |  |  |  |
|  |  |  | F_1626_-M |  |  |  |  |  |
|  |  |  | R_1629_-K |  |  |  |  |  |
|  |  |  | I_1649_-L |  |  |  |  |  |
|  |  |  | V_1655_-T |  |  |  |  |  |
|  |  |  | K_1658_-R |  |  |  |  |  |
|  |  |  | K_1659_-R |  |  |  |  |  |
|  |  |  | F_1674_-L |  |  |  |  |  |
