# Supplementary material for: A single mutation in Crimean-Congo hemorrhagic fever virus discovered in ticks impairs infectivity in human cells
Source: eLife. 2020 Oct 21;9:e50999. doi: 10.7554/eLife.50999 (PMC7652417; doi:10.7554/eLife.50999)
Supplement: Supplementary file 3. [file elife-50999-supp3.docx]

**Supplementary File 3.** Cell culture passage history of CCHFV strains pertinent to this study

| **CCHFV strain** | **Number of passage in cells** | **Cell line used** | **Number of Passage in new born mice** | **Lineage** | **Tick species origin** | **Country** |
| --- | --- | --- | --- | --- | --- | --- |
| IbAr10200 | 2 | VeroE6, Sw13 | 13 | Africa 3 | *H. excavatum* | Nigeria |
| MT-1302 | 0 | n/a | 0 | Europe 2 | *R. bursa* | Bulgaria |
| MT-1303 | 0 | n/a | 0 | Europe 2 | *R. bursa* | Bulgaria |
| MT-1303 | 0 | n/a | 0 | Europe 2 | *R. bursa* | Bulgaria |
| Pentalofos | 1 | VeroE6 | 0 | Europe 2 | *R. bursa* | Greece |
| AP92 | 1 | VeroE6 | 7 | Europe 2 | *R. bursa* | Greece |
| Cáceres 2014 | 0 | n/a | 0 | African 3 | *H. lusitanicum* | Spain |
| MG418 | 0 | n/a | 0 | Europe 2 | *H. marginatum* | Turkey |
