# Supplementary material for: A single mutation in Crimean-Congo hemorrhagic fever virus discovered in ticks impairs infectivity in human cells
Source: eLife. 2020 Oct 21;9:e50999. doi: 10.7554/eLife.50999 (PMC7652417; doi:10.7554/eLife.50999)
Supplement: Supplementary file 4. [file elife-50999-supp4.docx]

**Supplementary File 4.** Primers used to generate chimeric and point mutant IbAr10200 and MT-1303 GPC expression constructs

| **Primer name** | **Sequence** | **Description** |
| --- | --- | --- |
| pCAGGS_F | CGTGTGACCGGCGGCTCTAG | Primer in pCAGGS backbone to amplify N-term fragment of pCAGGS-GPC insert |
| pCAGGS_R | AAAAAGATCTGCTAGCTCGA | Primer in pCAGGS backbone to amplify C-term fragment of pCAGGS-GPC insert |
| MTGc_F | TGGTCTCTACCAGTGCCGTGGAAATGGAGAACCTGCCAGCC | Primer to amplify Gc region of MT-1303 for assembly into FLAG/HA-IbAr10200 Gn/MT-1303 Gc |
| 10200Gn_R | CACGGCACTGGTAGAGACCA | Primer to amplify Gn region of IbAr10200 for assembly into FLAG/HA-IbAr10200 Gn/MT-1303 Gc |
| MTGn_R | CACTGCGGTACTAGAAGTGA | Primer to amplify Gn region of MT-1303 for assembly into FLAG/HA-MT-1303 Gn/IbAr10200 Gc |
| 10200Gc_F | TCACTTCTAGTACCGCAGTGGAGATGGAAAATCTGCCCGC | Primer to amplify Gc region of IbAr10200 for assembly into FLAG/HA-MT-1303 Gn/IbAr10200 Gc |
| 10200_R1105G_F | GGTGAGTGGAGGGAGTGAAT | Site-directed mutagenesis primer to generate FLAG/HA-IbAr10200 GPC-R1105G |
| 10200_R1105G_R | ATTCACTCCCTCCACTCACC | Site-directed mutagenesis primer to generate FLAG/HA-IbAr10200 GPC-R1105G |
| 10200_R1104K_F | GGTGAGTGGAAAGAGTGAAT | Site-directed mutagenesis primer to generate FLAG/HA-IbAr10200 GPC-R1105K |
| 10200_R1105K_R | ATTCACTCTTTCCACTCACC | Site-directed mutagenesis primer to generate FLAG/HA-IbAr10200 GPC-R1105K |
| 10200_I1118M_F | GAACCGGCATGTCTTGGGAC | Site-directed mutagenesis primer to generate FLAG/HA-IbAr10200 GPC-I1118M |
| 10200_I1118M_R | GTCCCAAGACATGCCGGTTC | Site-directed mutagenesis primer to generate FLAG/HA-IbAr10200 GPC-I1118M |
| MT_G1116R_F | GGTGAGCGGGAGATCAGAGA | Site-directed mutagenesis primer to generate FLAG/HA-MT 1303 GPC-G1116R |
| MT_G1116R_R | TCTCTGATCTCCCGCTCACC | Site-directed mutagenesis primer to generate FLAG/HA-MT 1303 GPC-G1116R |
| MT_M1129I_F | GAACCGGCATCAGCTGGAAT | Site-directed mutagenesis primer to generate FLAG/HA-MT 1303 GPC-M1129I |
| MT_M1129I_R | ATTCCAGCTGATGCCGGTTC | Site-directed mutagenesis primer to generate FLAG/HA-MT 1303 GPC-M1129I |
| MT_G1116K_F | CAGAGAGCATCATGAAGCTG | Site-directed mutagenesis primer to generate FLAG/HA-MT 1303 GPC-G1116K |
| MT_G1116K_R | GCTTCATGATGCTCTCTGATTTCCCGCTCACCAGGATCTT | Site-directed mutagenesis primer to generate FLAG/HA-MT 1303 GPC-G1116K |
